# Building Emotional Awareness and Mental Health (BEAM): an open-pilot and feasibility study of a digital mental health and parenting intervention for mothers of infants

**DOI:** 10.1186/s40814-023-01245-x

**Published:** 2023-02-18

**Authors:** E. Bailin Xie, Makayla Freeman, Lara Penner-Goeke, Kristin Reynolds, Catherine Lebel, Gerald F. Giesbrecht, Charlie Rioux, Anna MacKinnon, Shannon Sauer-Zavala, Leslie E. Roos, Lianne Tomfohr-Madsen

**Affiliations:** 1 Department of Psychology, University of Calgary, Calgary, AB Canada; 2 Department of Educational and Counselling Psychology, and Special Education, University of British Columbia, Vancouver, BC Canada; 3 Department of Psychology, University of Manitoba, Winnipeg, MB Canada; 4 Alberta Children’s Hospital Research Institute (ACHRI), Calgary, AB Canada; 5 Department of Radiology, University of Calgary, Calgary, AB Canada; 6 Department of Pediatrics, University of Calgary, Calgary, AB Canada; 7 Department of Psychology, University of Oklahoma, Norman, OK USA; 8 Department of Psychiatry and Addictology, Université de Montréal, Montréal, QC Canada; 9 CHU Sainte-Justine Research Center, Montréal, QC Canada; 10 Department of Psychology, University of Kentucky, Lexington, KY USA; 11 Children’s Hospital Research Institute of Manitoba, Winnipeg, MB Canada

**Keywords:** Parenting, Maternal, Mental health, Depression, Pilot study

## Abstract

**Background:**

Maternal mental health concerns and parenting stress in the first few years following childbirth are common and pose significant risks to maternal and child well-being. The COVID-19 pandemic has led to increases in maternal depression and anxiety and has presented unique parenting stressors. Although early intervention is crucial, there are significant barriers to accessing care.

**Methods:**

To inform a larger randomized controlled trial, the current open-pilot trial investigated initial evidence for the feasibility, acceptability, and efficacy of a newly developed online group therapy and app-based mental health and parenting program (BEAM) for mothers of infants. Forty-six mothers 18 years or older with clinically elevated depression scores, with an infant aged 6–17 months old, and who lived in Manitoba or Alberta were enrolled in the 10-week program (starting in July 2021) and completed self-report surveys.

**Results:**

The majority of participants engaged in each of the program components at least once and participants indicated relatively high levels of app satisfaction, ease of use, and usefulness. However, there was a high level of attrition (46%). Paired-sample *t*-tests indicated significant pre- to post-intervention change in maternal depression, anxiety, and parenting stress, and in child internalizing, but not externalizing symptoms. Effect sizes were in the medium to high range, with the largest effect size observed for depressive symptoms (Cohen’s *d* = .93).

**Discussion:**

This study shows moderate levels of feasibility and strong preliminary efficacy of the BEAM program. Limitations to program design and delivery are being addressed for testing in adequately powered follow-up trials of the BEAM program for mothers of infants.

**Trial registration:**

NCT04772677. Registered on February 26 2021.

## Key messages regarding feasibility

- This is the first study to determine the feasibility and acceptability of a multimodal digital intervention tailored to the needs of parents of infants with elevated depression symptoms.
- Participants favourably rated the program App interface, as well as their satisfaction and perceived usefulness of the program. Although participant recruitment was successful and engagement was promising, attendance rates in group telehealth sessions were low and attrition rates were high. Findings suggested the need for program and trial design updates to promote more seamless app registration and use, participation in the forum, attendance at telehealth groups, and completion of follow-up questionnaires.
- Future trials will implement strategies to improve participant retention including program orientation meetings, streamlining the participant enrollment process, and curating a more flexible telehealth schedule that better suits family needs.

## Introduction

Postpartum mental health concerns are common [1–5] and pose significant long-term risks to maternal and child well-being. For mothers, depression contributes to elevated physical and psychological health problems and lower quality of life [6–10]. Furthermore, children exposed to maternal depression during their first 5 years of life have a higher risk of cognitive impairments, alterations to their physiological regulation, developmental delays, asthma, injury risk, and a high risk of developing mental illness in the course of their life [11–13]. Mothers with mental health concerns also report persistently high parenting stress [14–17], which is associated with negative parenting behaviours (i.e. harsh discipline and inappropriate expectations); delayed child cognitive, language, and motor development; and higher levels of child internalizing and externalizing problems [18–24].

During the COVID-19 pandemic, the prevalence of depression symptoms in pregnancy and the postpartum increased around the world [25–29], which is expected to lead to long-term impacts on parent-child relationships, parenting quality, and parent and child health outcomes in at least a subset of exposed families [30–32]. COVID-19 has also been associated with unique parenting stressors related to the deprivation of family support and altered family relationships [33]. For mothers with depression, additional stressors, such as those experienced during COVID-19, have been shown to worsen depression severity and symptoms and increase the risk of elevated parenting stress persisting throughout their child’s early years [14, 30, 34].

Early intervention is crucial to prevent long-term adverse consequences of mental health symptoms for mothers and their children. Without treatment, maternal depressive symptoms tend to persist throughout the preschool years [35] and the negative consequences of maternal mental illness are most pronounced when depression persists [13, 36]. Although evidence-based treatments exist to address maternal mental illness, there are significant barriers to accessing care, which have worsened as a result of the COVID-19 pandemic. Prior to the pandemic, birthing parents in the perinatal period identified lack of childcare and lack of time as barriers to seeking mental health services [37]. These barriers were particularly pertinent during the COVID-19 pandemic due to overwhelming childcare demands, physical isolation from social and support networks, unemployment and financial strain, and closure of existing services [38].

E-health interventions offer a promising avenue to address the barriers to traditional healthcare services. It is estimated that in 2018, 91.3% of Canadians 15 years and older use the Internet and 88.1% have a smartphone at home. These estimates increase to 98.2% of Canadians who use and have access to Internet and 97.1% who have a smartphone when examining individuals between the ages of 24 and 44 [39, 40]. These statistics were similar across the two Canadian provinces included in the current study, with 94.1% and 91.2% of people using Internet in Alberta and Manitoba, respectively [41]. Previous research has found that mothers with perinatal mental health problems indicate web-based resources are a preferred way to access mental health support [37]. As such, app-based mental health interventions are a potential avenue to reach a larger number of parents with mental health problems and early results are promising. A meta-analysis found that smartphone mental health interventions reduced symptoms of depression [42] compared to both active and inactive control conditions.

However, although digital interventions can increase flexibility and convenience for health care delivery, issues of inequality can emerge due to concerns with the distribution of digital technologies with some already disadvantaged groups reporting more barriers to accessing eHealth [43]. These barriers can include less access to reliable Internet, computers or smartphones, or a private space in the home to participate in sensitive telehealth sessions [43].

Furthermore, there are significant issues when using app-based interventions related to engagement and retention of participants. Downloading an intervention app has been found to be a significant challenge for individuals experiencing depression, specifically. In one clinical trial for adults with mild to moderate depression, less than half of the participants who were instructed to download an intervention App did so [44]. In an analysis of real-world mental health app usage, a study found that very few users (median= 4%) opened their mental health app daily [45]. Poor usability, unfriendly design, lack of trust, and concerns about privacy are specific factors that may lead to low user engagement with mental health apps [46].

There is evidence to suggest that the inclusion of a peer support component in app designs can increase participants’ app engagement [45] and including a peer support in mental health treatment can lead to more satisfaction with treatment of postpartum depression [47]. Individuals have expressed appreciation for peer support during the perinatal period with one study finding that peers were the preferred delivery agents of maternal psychosocial intervention for perinatal mental health concerns [48] and a recent survey finding that 85% of parents would prefer connecting with peers if participating in a digital program [49]. Support from other birthing parents has been found to be particularly important in the recovery of postpartum depression and including peer support in traditional mental health treatment is associated with greater levels of satisfaction in the treatment of postpartum depression [47]. This is unsurprising as individuals in the perinatal period often rely on social support in the postnatal period to care for their infants, resume daily activities, and recover from childbirth [50]. However, according to systematic review findings, there is often a lack of social support in apps that target individual mental health [45].

In addition to the limitations of previous digital interventions targeting mental health, there has been little evidence for the efficacy of existing digital interventions targeting parenting practices on parent and child outcomes. A meta-analysis suggests small-moderate effect sizes of digital parenting interventions, with larger effects observed when programs include interactive platforms with a therapist instead of being completely self-led [51]. Overall, the vast majority of digital programs have been unimodal, targeting either mental health or parenting. With evidence suggesting that multimodal programs that target both mental health and parenting are most promising for improving parent, child, and family well-being [36], there is a need for the development of effective multimodal digital interventions tailored to the needs of parents with mental health symptoms.

In response to these limitations of previous digital programs, our group has developed the Building Emotional Awareness and Mental Health (BEAM) program, a 10-week app-based intervention that provides mothers with mental health and parenting psychoeducation, a peer support forum, and weekly e-health group therapy sessions. This open-pilot trial investigated initial evidence for the feasibility, acceptability, and efficacy of the BEAM program when delivered to mothers of infants aged 6–17 months to help inform a larger randomized controlled trial. It was hypothesized that participation in the BEAM program would be associated with decreased maternal depression and parenting stress (primary outcomes), as well as reduced maternal anxiety and child internalizing and externalizing symptoms (secondary outcomes) from pre- to post-intervention.

## Method

### Participants and procedure

Eligible individuals were people who identified as mothers or as a female primary caregiver and who were 18 years or older with a 6–17-month-old child. Participants had to live in Manitoba or Alberta, Canada, and report a clinically elevated score of depression (≥ 10 on the Patient Health Questionnaire; PHQ-9) [52]. Eligible participants also needed to be available for weekly Zoom sessions. Participants who reported a history of attempted suicide in the past year, self-harm in the past 6 months, or significant suicidal ideation were excluded from the BEAM program, since the program was not considered suitable to treat these mental health needs. These participants received a list of alternative mental health resources. Participants were recruited through online platforms (i.e. lab Facebook, Instagram, Twitter, and website), and through community partner agencies. Recruitment posters and photos, with a link to our eligibility screener, were advertised on social media and circulated via email. Eligible mothers received up to $70 compensation (in Amazon gift cards) for participating in the BEAM program.

All eligible mothers completed a survey between 4 and 8 weeks prior to the program start date (which will be referred to as “pre” hereafter) and a survey 3–6 weeks after the end of the program (which will be referred to as “post” hereafter). Data collection was conducted online using self-report surveys collected using Research Electronic Data Capture (REDCap). Socio-demographic characteristics of the sample are presented in Table 1.

**Table 1:** Participant demographics

Baseline characteristics (***n*** = 46)	Value
Age of mothers (years; M, SD)	30.83 (4.86)
Age range	19–39
Household income > 90K CAD (%)	42.9
Ethnicity (mother; %)	
European Canadian	69.5
Indigenous	17.4
Asian	8.6
Other	4.3
Maternal education (%)	
Less than high school	4.3
High school	13
College/technical school	28.3
Bachelor’s degree	30.4
Graduate or professional degree	23.9
Married/common law (%)	93.5
Community type (%)	
Large city	50
Town or small city	38.6
Rural area	11.4
Employment status (%)	
On leave	53.3
Part or full-time work	33.4
Number of children (%)	
1	60.9
2	23.9
3+	15.2
Age of child (months; M, SD)^a^	11.23 (3.32)
Age range (months)	6–17

*M* Mean, *SD *Standard deviation, *CAD *Canadian dollar

^a^Child refers to the child that is participating in BEAM (i.e. the oldest child within the 6–17-month range)

### Intervention—BEAM

The content from the BEAM program was delivered via mobile application and was supported by weekly group zoom sessions led by psychologists and clinical psychology trainees (Masters and Doctoral students). The BEAM app is a 10-week program that includes weekly 15–30-min video sessions on mental health and parenting as well as closed community forums that participants use to connect and communicate with each other and with parent and support coaches (described below).

#### Psychoeducation

The BEAM program includes a psychoeducation component which is comprised of video modules about mental health and parenting. Most weeks consisted of two videos (one mental health video and one parenting video) that were each approximately 10 min in length (ranging from 6 to 14 min). Mental health videos provided information and tools following the principles of the Unified Protocol, a best-practice program based on cognitive-behavioural principles, to address dysregulated emotions across mood and anxiety disorders [53, 54]. Supportive parenting videos provided mothers with behaviour management strategies and were designed to help parents understand their children’s challenging behaviours and promote positive parent-child interactions. An overview of the weekly video content is presented in Table 2. Activities were available each week and were designed to encourage participants to reflect and practice what they learned. Participants were also encouraged to engage in meaningful and interactive discussions related to the weekly topics with a supportive person in their life and with their BEAM community on the forum.

**Table 2:** Overview of weekly telehealth video content

Week	Mental Health	Parenting
1	Monitoring mental health and identifying personal values	Parenting myths and identifying parenting values
2	Goal setting and maintaining motivation	Finding moments of joy to improve parent-child relationships
3	Emotional response and how emotions work	Regulating your child’s emotions
4	Walking through emotional experience (ARC)	Using routines to help regulate your child’s emotions
5	Mindful Emotion Awareness	Mindfully engaging with your child
6	Thinking patterns and cognitive flexibility	Finding flexibility to help navigate tough situations with your child
7	Countering emotion-driven behaviours with alternative actions	Managing your child’s big emotions
8	How physical sensations impact emotional experiences and how to face them	Staying present when your child has big emotions
9	Emotion exposures to improve coping	Skills for you and your child to cope with tantrums
10	Program takeaways; evaluating and maintaining progress	Feeling well and balanced as a parent

#### Telehealth groups

Weekly zoom telehealth groups provided an opportunity for clinical coaches (MA, PhD, or clinical psychology trainees) to review weekly content and facilitate conversations among participants. Participants had the opportunity to discuss material with their clinical coach and other mothers in the BEAM program, ask questions, and gain a better sense of community. Including facilitator contact to promote human support has been found to help enhance the effectiveness of and adherence to e-health interventions [55–57]. Contact with facilitators is proposed to increase program adherence through the sense of accountability to a coach who is viewed as having expertise and being trustworthy and benevolent [56]. Weekly telehealth sessions occurred at the same time each week. The time of the session was determined based on the time which most participants reported being available for.

#### Online community forum

The program included online community forums which were designed to encourage mothers to continue to build social connections with other mothers in the program outside of the weekly telehealth groups. The forums were moderated by clinical and parent coaches (described below). There were intervention forums which included semi-structured discussion topics related to the weekly video content. For example, mothers discussed how the weeks went and their experience implementing the weekly topics into their daily lives. There was also a support forum that consisted of open-ended discussions and provided a space for mothers to post questions to their peers and coach and share anecdotes and/or photos of their wellness journey. Participants were also invited to ask mental health and parenting questions on an “Ask the Expert” forum, which was monitored and responded to by clinical coaches.

#### Coaches

*Parent coaches* were mothers who had recently completed another research lab-implemented group-based intervention for their own mental health needs (at the University of Manitoba) and who were interested in being a part of the BEAM community to promote the mental wellness of mothers. The parent coach provided support for the mothers in the program and participated in the community to promote discussion and monitor the content posted on the forum. *Clinical coaches* were MA, PhD, or student clinicians who were responsible for responding to questions about group content and responding to clinically sensitive content on the forum. Clinical coaches conducted risk assessments in a one-to-one email or phone check-in when participants expressed significant distress on the forum, in group, or in weekly survey responses.

### Measures

#### Socio-demographic variables

Demographic information was collected via a self-report questionnaire administered as part of the baseline questionnaire. Demographic variables collected included maternal age, ethnicity, education level, employment status, community type, marital status, medication and service use in the past month, parity, age and sex of child, and household income.

### Primary outcomes

#### Feasibility and acceptability

The mHealth App Usability Questionnaire (MAUQ) [58] was used to assess participants’ perception of the app usability after program completion. The MAUQ contains 18 items assessing three subscales: (1) app’s ease of use, (2) interface and satisfaction, and (3) usefulness. Items were measured on a 7-point Likert scale ranging from 1 (disagree*)* to 7 (agree), with higher scores indicating better usability of the app. The MAUQ has been found to have excellent internal reliability (*α* = 0.914) [58]. Questions were also developed and used for the pilot study to assess the level of participant engagement across different components of the BEAM program (i.e. video modules, forum, and telehealth groups). Participants were asked about whether they had engaged in each component (e.g. “Did you ever participate in the forums?”) and the amount of engagement in each component (e.g. “How many videos did you watch?”). Measures of recruitment, enrollment, and retention were also included to assess participant interest in the BEAM program and the acceptability of the run-in process involved.

#### Depression symptoms

Symptoms of depression were assessed at the participant eligibility screener and at the post-questionnaire using the Patient Health Questionnaire (PHQ-9) [52]. The PHQ-9 is a self-administered 9-item questionnaire that assessed the presence (PHQ-9 ≥10) and severity of depressive symptoms: 0–4 (minimal or none), 5–9 (mild), 10–14 (moderate), 15–19 (moderately severe), and 20–27 (severe). The established PHQ-9 cutoff score (PHQ-9 ≥10) has been shown to demonstrate high sensitivity and specificity in detecting depression in a perinatal population [59].

#### Parenting stress

The Parenting Stress Index—Short Form (PSI/SF) was administered to participants at pre- and post-intervention. The PSI is a self-administered 36-item scale that measures parent-reported stress and interactional style along 3 domains: how parents feel in their role, how satisfied they are in the relationship with the child, and how difficult they perceive their child to be [60]. Parents reported their level of agreement to the scale items on a 5-point Likert scale (1=strongly disagree; 5=strongly agree), which were summed to produce a total stress score. Total scores range from 36 to 180, with higher scores indicating higher parenting stress. The PSI has been demonstrated to have high internal consistency and is a useful assessment tool for clinical interventions for parents of young children [61–64].

### Secondary outcomes

#### Anxiety symptoms

Symptoms of general anxiety were assessed at pre- and post-intervention using the Generalized Anxiety Disorder 7-Item Scale (GAD-7) [65]. The GAD-7 is a self-administered scale with 7 items that are rated on a 4-point Likert scale (0 = not at all, 3 = nearly every day). Sum scores range from 0 to 21, with higher scores indicating more severe anxiety symptoms. The following recommended cutoff scores were used: 10–14 (moderate) and 15 (severe anxiety). The GAD-7 has been shown to be a clinically useful instrument in perinatal populations [66].

#### Child mental health and behaviour

Child mental health and behaviour were assessed at pre- and post-intervention using the Child Behaviour Checklist (CBCL) [67]. The CBCL is a parent-report questionnaire that measures child mental health and behaviour challenges using 113 questions scored on a 3-point Likert scale (0=problem behaviour is absent; 1=occurs sometimes; 2=occurs often). Internalizing and externalizing subscale scores were computed. The internalizing subscale includes 36 items, with possible subscale scores ranging from 0 to 72 and the externalizing subscale includes 23 items, with possible subscale scores ranging from 0 to 46. The CBCL is one of the most widely used measures of child mental health and behaviour worldwide [68, 69], has demonstrated good reliability and validity [70], and has been established as a sensitive and efficient tool for assessing child psychiatric disorders [69]. The CBCL 1^1^/_2_ –5 was used, which is commonly used for children ages 18 months and older [71, 72].

### Statistical analyses

All analyses were conducted using IBM SPSS Statistics 28.0. A series of paired-sample *t*-tests were conducted to analyse whether participants experienced changes in their mental health and parenting stress as well as their children’s internalizing and externalizing challenges [73]. Cohen’s *d* statistic was used to measure effect sizes, with 0.20, 0.50, and 0.80 representing small, medium, and large effect sizes, respectively [74]. Clinically significant change in mental health symptoms was assessed using a binary variable for whether participants achieved the minimum point reduction on the depression and anxiety variables from T1 to T2 (i.e. 5-point and 4-point reductions on the PHQ-9 and GAD-7, respectively).

## Results

### Feasibility and acceptability

**Fig. 1:**
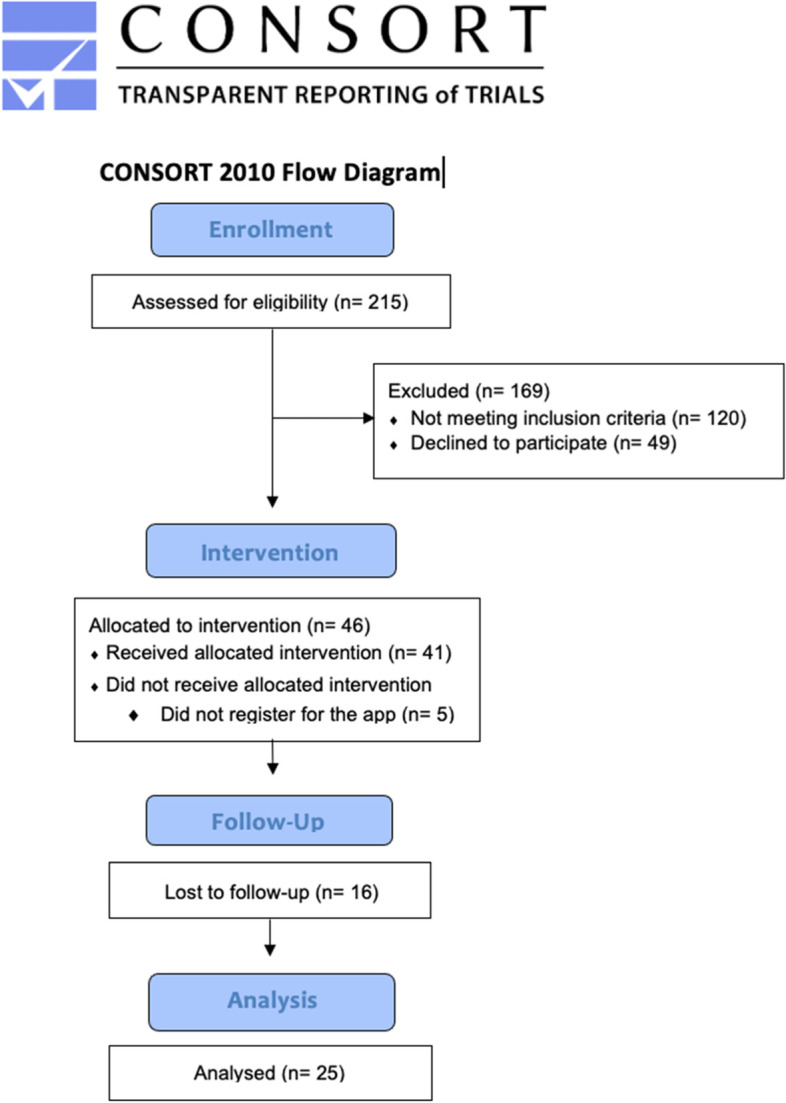
Consort flow diagram *Note.* Individuals lost to follow-up refer to those who did not complete post-intervention questionnaires

The target sample size was 40–45 participants. As planned, recruitment targets were reached within 5 weeks. Two hundred and fifteen mothers completed the eligibility screener from May to June 2021. One hundred and twenty mothers were excluded due to ineligibility based on screener responses. If mothers were eligible, they were contacted by our research coordinator via email with more information about the program and research process. At this point, participants were informed that the program would begin in June 2021. Forty-nine mothers either declined to participate or failed to respond to our email and were therefore excluded. Of the participants who completed the eligibility screener, 46 (21%) were eligible, consented to participation, and were enrolled in the BEAM program. After enrollment, mothers were required to provide a preferred login and password to our project coordinator and register with their phone number and address on our clinical electronic record-keeping system. Five participants did not provide this information and therefore were not registered for the app and never accessed the intervention. Forty-one mothers were successfully registered on the app. Due to several technological delays in the app launch, the actual program’s start date was mid-July 2021. Sixteen participants did not complete the T2 post-questionnaires and were excluded from the analysis. Twenty-three mothers completed all post-program measures and two had partial completion of the post-questionnaire, leaving a complete sample of *n* = 25 (Fig. 1).

The enrollment process was limited by a potentially complex number of steps (e.g. requesting that participants emailed the research team following an eligibility screener to confirm interest) and failing to ensure that participants were interested in downloading the App and attending telehealth groups. Independent sample *t*-tests and chi-square tests were conducted to determine whether there were any significant differences in baseline characteristics or sociodemographic variables between participants based on completion status. No significant group differences emerged (*p* = .068–.943).

In terms of program engagement^1^, 82.6% watched at least one video and participated in the forum at least once. Of the participants who watched at least one video, 31.6% of participants watched 1–5 videos, 31.6% watched 6–10 videos, 15.8% watched 11–15 videos, and 21.1% watched 16+ videos out of the total 31 videos. Eighty-seven percent of participants attended at least one telehealth group session. Of the participants who attended telehealth group(s), 30% attended 1–3 sessions, 30% attended 4–6, 15% attended 7–9, and 25% attended 10+ sessions. The number of people who attended the telehealth groups fluctuated between 7 (week 6) and 22 (week 1) during the 10 weeks of scheduled sessions. There did appear to be a decline in attendance from the beginning to the end of the program. Participant-reported barriers to telehealth session attendance included family commitments, work commitments, energy levels, time of day, and hesitancy around the perceived usefulness of group therapy. On the forum, participant-reported use ranged from one time to approximately 15 times on the forum. Forum topics discussed included child sleep and behavioural challenges, strategies for navigating difficult partner and family relationships, and successes and challenges in applying that week’s program content. Participant-reported barriers to forum engagement included lack of time and discomfort in sharing personal information.

In terms of social support, 60.9% of respondents rated ≥ 4 on a 6-point scale (with higher values indicating greater agreement) on an item that asked whether participants agreed with the following statement: “The BEAM program was a good source of social support for me.” Results from the MAUQ questionnaire indicated relatively high levels of ease of use, app satisfaction, and usefulness. Most participants rated ≥ 5 on the following items: “The app was very easy to use” (76.1%), “It was easy for me to learn to use the app” (85.8%), “Overall, I am satisfied with this app” (71.4%), and “The app would be useful for my health and well-being” (63.6%). See Table 3 for the descriptive statistics for the engagement questions and MAUQ subscales.

**Table 3:** Participant engagement and BEAM app usability

Program engagement	*n* (%)^a^	Range
Watched at least 1 video	19 *(82.6)*	1–16+
Participated in the forum at least once	19 *(82.6)*	1–15
Attended at least 1 telehealth session	20 *(87)*	1–10+
MAUQ	*M* (SD)	Range
Ease of use	27.43 (*7.12*)	7–35
Interface and satisfaction	35.09 (*10.64*)	8–49
Usefulness	28.55 (9.45)	6–42

*Note. MAUQ*, mHealth App Usability Questionnaire

^a^Out of 23 respondents. For the MAUQ ease of use subscale, 2 participants had partial completion (*n* = 21)

### Program efficacy

Paired-sample *t*-tests (see Table 4) indicated significant pre- to post-intervention change in maternal mental health symptoms (depression and anxiety), parenting stress, and child internalizing, but not externalizing, problems. The effect size was large for maternal depressive symptoms and medium for parenting stress, maternal anxiety symptoms, and child internalizing symptoms. With regard to clinical significance, 60% of participants had clinically significant change in depression symptoms (≥5-point reduction on the PHQ-9) and 41.7% of participants had clinically significant change in anxiety symptoms (≥4-point reduction on the GAD-7).

**Table 4:** Pre, post, and change scores for program efficacy outcomes

	Pre *M* (SD)	Post *M* (SD)	Change *M* (SD)	Within subject *t*-test	Effect-size
Primary outcomes					
Maternal depression	15.96 (3.88)	10.17 (6.36)	5.80 (6.23)	*t*(24) = 4.65, *p* <.001	*d* = .93
Parenting stress	92.19 (20.22)	83.37 (19.64)	8.82 (14.07)	*t*(24) = 3.13, *p* = .002	*d* = .63
Secondary outcomes					
Maternal anxiety	13.85 (5.05)	10.22 (5.58)	6.36 (6.21)	*t*(23) = 2.86, *p* = .004	*d* = .58
Child internalizing	8.34 (7.18)	6.19 (5.05)	2.14 (4.19)	*t*(21) = 2.40, *p* = .013	*d* = .51
Child externalizing	11.22 (6.46)	12.75 (6.84)	−1.52 (5.85)	*t*(21) = −1.22, *p* = .118	*d* = −.26

## Discussion

Prior to conducting a larger randomized controlled trial, the current small and open pilot study investigated initial evidence for the feasibility, acceptability, and efficacy of the BEAM program when delivered to mothers of infants. Results of the current study support moderate levels of feasibility and acceptability of the BEAM program based on recruitment, retention rates, and participants’ self-reported usability and satisfaction with the program. There was preliminary evidence for the efficacy of the BEAM program when delivered to mothers of infants based on the statistically significant reductions in key program outcomes (i.e. mental health, parenting stress, and child internalizing problems) and moderate support for clinically significant reductions in maternal mental health concerns.

### Program efficacy

As hypothesized, participants reported reductions in depression symptoms (*d* = .93), parenting stress (*d* = .63), anxiety (*d* = .58), and infant internalizing problems (*d* = .51). Compared to recent meta-analyses of studies testing interventions (traditional psychotherapy and telehealth) for maternal mental health in the postpartum period, our study found similar or slightly greater effect sizes in terms of reductions in depression and anxiety symptoms, infant internalizing problems, and parenting stress [75–77]. These findings are promising as maternal mental health in early childhood has important implications for child behaviour and executive functioning in later childhood [78]. Furthermore, treating maternal depressive symptoms in early childhood can have long-term benefits for their child as persistent depressive symptoms have been found to predict child behaviour problems in early adolescence above and beyond postpartum depression alone [79].

The observed reduction in infant internalizing symptoms is also promising as addressing internalizing symptoms through interventions in early childhood has been shown to yield long-term preventative benefits for their internalizing symptoms throughout childhood [80]. The reductions in internalizing symptoms may have been attributable to addressing various risk and protective factors for child internalizing symptoms, increasing parenting skills and self-efficacy, and promoting child emotion regulation [80]. Although evidence suggests that digital-based parent training can be helpful for improving child disruptive behaviour problems [81], the non-significant effect on child externalizing symptoms in the current study may have been due to the use of the CBCL for a very young sample. Although these promising preliminary efficacy findings support further testing, future efficacy trials with more rigorous study designs are needed to better understand these associations.

### Feasibility and acceptability

Although recruitment goals were reached within 5 weeks, there was a high level of attrition (i.e. individuals who did not register for the app or did not complete post-intervention questionnaires; 46%). Furthermore, telehealth group attendance was lower than expected, which may have been impacted by difficulties related to determining a suitable time for telehealth sessions across multiple provinces (e.g. dinner and bedtime interferences). For instance, some participants shared conflicts with needing to care for and put children to bed during this time. In addition, due to delays with app development, the program ran over the summer months, a time when many families were away on vacation and were not able to attend groups. To help reconcile low attendance rates, a 4-week break was introduced halfway through the program (between weeks 5 and 6). During this break, there were “expert talks” on topics of interest (e.g. self-compassion and sleep). However, it is possible that this break impacted treatment outcomes, attrition, and/or satisfaction with the intervention. For future trials, groups will occur at various times throughout the day and participants will be provided with the choice that is most suitable. Furthermore, trials will not occur in the summer months.

Participants who completed post-intervention questionnaires favourably rated app interface and satisfaction as well as usefulness. Although this is consistent with previous work showing high user satisfaction in mental health app-based interventions, review findings have shown that many previous evaluations of mobile mental health apps have used non-standardized measures of usability and satisfaction, potentially inflating these reported ratings [82]. A strength of this study is the use of a standardized measure of user satisfaction (i.e. MAUQ; [58]). Furthermore, the majority of participants who completed post questionnaires reported engaging in each of the program components at least once (i.e. forum, video modules, telehealth groups). These results are promising as engagement in app-based interventions for depression has been found to be consistently low [83]. Promoting app registration and use, participation in the forum, attendance at telehealth groups, and completion of questionnaires remain important areas of improvement for future studies.

### Strengths, limitations, and future directions

Strengths of the BEAM program include the combination of both parenting and mental health content, which can lead to greater treatment effects compared to those that target either one or the other, as supported by our medium-to-large effect sizes. Targeting parental mental health, and equipping parents with evidence-based parenting strategies can improve health outcomes for both parents and their young children [13, 36]. The BEAM program also included a number of interactive components which provided participants with the ability to engage in different components of the program, depending on their needs and preferences. Strategies such as engagement reminder emails and app notifications were also incorporated, which have been found to contribute to greater mental health benefits from e-health interventions [84, 85] and increase the effectiveness of online parenting programs [86]. Furthermore, the digital delivery of the BEAM program helps to circumvent birthing parents’ identified barriers to traditional mental health services, including lack of childcare [37]. Lastly, the inclusion of a parent advisory board (i.e. mothers who previously participated in group mental health and parenting interventions themselves) during the development and delivery of the intervention was a strength of the current study. Asking for ongoing feedback from the parent advisory board helped to ensure that the BEAM program is addressing the specific needs of mothers of young children.

It is possible that challenges regarding program design and program delivery and the resulting trial design limitations contributed to the high attrition rate and ratings of app usability and acceptability. During the app development process, there were challenges in communication with the digital design company (see [87]). Challenges included obtaining PHIA-compliant server storage needed for security purposes and the need to drop expected features and implement alternative features due to budget constraints, which delayed the launch of the intervention by one month after the anticipated launch date. Although participants were informed of the initially planned start date, it is possible that the launch delay impacted participants’ ability or eagerness to participate in the programm (i.e. due to additional summer responsibilities; [87]). Furthermore, there were challenges related to app functionality including many participants being unable to access weekly videos directly from the App due to incompatibility with certain devices. Therefore, these participants had to access videos via YouTube links. There were also challenges related to program delivery (see [88]). For instance, attrition may have been impacted by complications related to being unable to contact participants if they did not respond with identified log-in information for App access. To circumvent these challenges for future trials, the research team has made adjustments to ensure the timely launch of the program and revisions such as obtaining participant contact information at the time of study enrollment and providing participants with a username and password for the App. To help address the challenges associated with assessing participant interest and enrollment, future trials will include program orientation meetings with interested participants and remove steps requiring participants to email the research team for enrollment purposes.

Furthermore, there was no control group in this open-pilot trial. Thus, it is possible that extraneous factors contributed to the observed reductions in mental health and parenting stress. For instance, COVID-19 lockdown protocols and restrictions were eased in mid-summer in Alberta and Manitoba, Canada, which may have impacted levels of mental health concerns and parenting stress. Furthermore, the time of year may have played a role as mental health has been found to improve during summer, compared to winter months [89]. Future planned efficacy trials will include a control group to account for these potential influences. Although the sample size was adequate for a pilot study, the sample size was too small to examine moderators of change.

The use of the CBCL to test child outcomes may have been a limitation due to the age of our sample at the date of recruitment (6–17 months). Although the CBCL has not specifically been validated for ages 6–17 months, there are limited scales of child outcomes that have been validated for this age group. Due to the longitudinal design of the current study where we were collecting data as children grew older, the CBCL was used as an exploratory measure of child outcomes. However, future efficacy trials will ensure the use of measures that are more appropriate for the sample age range.

Furthermore, although those who did not complete the post-intervention surveys did not statistically significantly differ from the completer sample in terms of baseline characteristics, it is possible that those who did not complete post-survey questionnaires did not experience the same benefits or perceive the same degree of usability or feasibility of the BEAM program. Future randomized controlled trials will use missing data handling strategies in the data analysis stage to help control for this possibility. Moreover, although this feasibility study did not correct for multiple comparisons due to the small sample size, future trials will implement a more robust statistical analysis plan that includes corrections for multiple comparisons [90].

Lastly, it is important to address issues of generalizability. This sample was largely European Canadian and of high socio-economic status. Parents with greater socio-economic stressors are at higher risk of parenting stress and depression [91, 92] and should be better represented in future research. Future trials will involve community agency collaboration to assist with a greater reach and a more representative sample. Furthermore, to help address the issues of inequalities that can emerge in the context of e-health interventions (e.g. greater barriers to access for under-resourced groups; [43]), future studies will continue to ensure that interested participants who do not have access to appropriate technology will be provided with a smartphone device to use for the duration of the program.

A pilot randomized controlled trial has also been conducted in an older child age range [88]. This pilot study tested whether the BEAM program was effective for mothers of toddlers, rather than infants, when compared to a treatment as usual control group. Using a rapid cycle iteration approach, involving the incorporation of pilot study feedback from both infant and toddler target ages and focus group participant interview content, improvements to the program have been made prior to additional testing. Larger efficacy trials that address the identified challenges and include longer-term follow periods are currently underway [93]. This will allow us to explore whether there are sustained beneficial effects of the BEAM program. Larger trials are including larger sample sizes to allow for the examination of moderators of change to better understand for whom this program is most beneficial as well as predictors of drop-out.

## Conclusions

This study found moderate levels of feasibility as well as strong preliminary efficacy of the BEAM program. Limitations to program design and delivery are being addressed for testing in adequately powered follow-up trials of the BEAM program for mothers of infants.

## Data Availability

The dataset analysed during the current study is available from the corresponding author on reasonable request.

## Footnotes

1 Statistics about program engagement were obtained from the 25 participants who completed post questionnaires.
